# Asthma Control and Its Relationship with Obstructive Sleep Apnea (OSA) in Older Adults

**DOI:** 10.1155/2013/251567

**Published:** 2013-11-06

**Authors:** Mihaela Teodorescu, David A. Polomis, Ronald E. Gangnon, Jessica E. Fedie, Flavia B. Consens, Ronald D. Chervin, Mihai C. Teodorescu

**Affiliations:** ^1^James B. Skatrud Pulmonary/Sleep Research Laboratory, Medical Service, William S. Middleton Memorial Veterans Hospital, Madison, WI, USA; ^2^Section of Allergy, Pulmonary and Critical Care Medicine, USA; ^3^Center for Sleep Medicine and Sleep Research/Wisconsin Sleep, University of Wisconsin School of Medicine and Public Health, Madison, WI, USA; ^4^Department of Biostatistics and Medical Informatics and Population Health Sciences, University of Wisconsin School of Medicine and Public Health, Madison, WI, USA; ^5^Department of Neurology and Sleep Disorders Center, University of Michigan Health System, Ann Arbor, MI, USA; ^6^Section of Geriatrics and Gerontology, University of Wisconsin School of Medicine and Public Health, Madison, WI, USA

## Abstract

*Background/Objectives*. Asthma in older individuals is poorly understood. We aimed to characterize the older asthma phenotype and test its association with obstructive sleep apnea (OSA). *Design*. Cross-sectional. *Setting*. Pulmonary and Asthma/Allergy clinics. *Participants*. 659 asthma subjects aged 18–59 years (younger) and 154 aged 60–75 (older). *Measurements*. Sleep Apnea scale of Sleep Disorders Questionnaire (SA-SDQ), asthma severity step (1–4, severe if step 3 or 4), established OSA diagnosis, continuous positive airway pressure (CPAP) use, and comorbidities. *Results*. Older versus younger had worse control, as assessed by asthma step, lung function, and inhaled corticosteroid use. Among older subjects, after controlling for known asthma aggravators, OSA diagnosis was the only factor robustly associated with severe asthma: on average, OSA was associated with nearly 7 times greater likelihood of severe asthma in an older individual (OR = 6.67). This relationship was of greater magnitude than in younger subjects (OR = 2.16). CPAP use attenuated the likelihood of severe asthma in older subjects by 91% (*P* = 0.005), much more than in the younger asthmatics. *Conclusion*. Diagnosed OSA increases the risk for worse asthma control in older patients, while CPAP therapy may have greater impact on asthma outcomes. Unrecognized OSA may be a reason for poor asthma control, particularly among older patients.

## 1. Introduction 

Asthma is a major health problem in the general population. As many patients develop asthma in childhood or adolescence, large community studies have focused on asthma in early years. While the prevalence is estimated to be 6.5–17% [1] and may be similar to that seen in younger adults, asthma is frequently underrecognized as a geriatric respiratory disorder and often remains undiagnosed [2]. This may be due to the fact that older adults tend to underreport symptoms, have limited subjective awareness, lack perception or attribution to pulmonary pathology [2], or lack access to lung function testing such as spirometry and peak flow [3].

Asthma-associated morbidity and mortality increase with older age [4]. The number of unscheduled ambulatory visits, emergency visits, and hospitalizations is high in elderly asthmatics [5]. Elderly individuals with asthma in comparison to young adults have 14-fold higher asthma-related death rates and are twice as likely to be hospitalized in a given year [6]. Death rates attributable to asthma increase exponentially after age of 65 [7], with women and particularly black women being the most affected [8]. Nonetheless, suboptimal therapeutic management has often been the norm [4], perhaps as a result of the poor recognition and understanding of this phenotype. Inhaled corticosteroids were historically underutilized, with deviation from recommended clinical guidelines. In addition, therapeutic decisions may have been hampered by the fact that many randomized treatment trials excluded patients older than 65 years, or those with substantial comorbidity [9].

Quality of sleep in older patients with asthma is poorer compared to those with chronic obstructive pulmonary disease (COPD) or chronic bronchitis [10]. Whereas some comorbid conditions, such as rhinitis and gastroesophageal reflux disease (GERD), are well recognized to aggravate asthma, little is known about the potential role of obstructive sleep apnea (OSA), another condition with high prevalence in the elderly [11]. Accumulating data in younger samples suggests a role of OSA in asthma control. Treatment of OSA with continuous positive airway pressure (CPAP) improves asthma symptoms, peak flow rates, and quality of life [12, 13]. The sleep disorder is also an important risk factor for frequent exacerbations in difficult-to-control asthma patients [14].

We therefore used data from a sizeable, ongoing survey [15] to examine whether clinically-diagnosed and untreated OSA predicts asthma control among older patients and to assess whether the magnitude of such associations resembles those found among younger patients. Our hypothesis was that OSA predicts worse asthma control in older patients, as it does in the young. Preliminary results of this work have been published in abstract form [16]. Subsets of these data have been analyzed on other aspects of the relationships between OSA and asthma, which were included in our prior reports [15–17].

## 2. Methods

### 2.1. Study Participants

The sample included asthma patients returning for routine follow-up visits at the University of Michigan Pulmonary Clinics and Asthma-Airways Center and at the University of Wisconsin Allergy and Pulmonary Clinics. Subjects were enrolled as part of a larger ongoing study on the relationship between OSA and asthma, including use of informed consent and approval from each Institutional Review Board. Data were collected between May 2004 and April 2006 (at the University of Michigan) and July 2007 and December 2009 (at the University of Wisconsin). As required by the design of the parent study, patients 18–75 years old who were able and willing to provide informed consent and complete our survey were invited to participate. All subjects had specialist-diagnosed and managed asthma [18]. Patients underwent history, physical examination, asthma control assessments, and spirometry with each clinic visit. Those at urgent asthma-related visits or pregnant women were not enrolled. 

### 2.2. Survey Content

The self-administered survey package, distributed by study assistants, is comprised of two questionnaires and additional questions on demographics. The first instrument, the Sleep Apnea scale of the Sleep Disorders Questionnaire (SA-SDQ) assesses OSA symptoms and summarizes its risk [19]. This scale consists of eight symptom items inquiring about loud snoring disruptive to the bed partner, cessation of breathing in sleep, sudden gasping arousals, worsening of snoring while supine or after alcohol, nocturnal sweating and nasal congestion, and history of hypertension. Responses are recorded on a 5-point Likert scale (“never” to “always”). Demographic data on weight, age, smoking, and body mass index (BMI) are rated on a 1 to 5 scale, with the overall SA-SDQ score ranging between 12 and 60. This scale was validated in a large sample of sleep patients [19].

The second survey instrument asked about frequency of daytime and nighttime asthma symptoms (as below) following the National Asthma Education and Prevention Program classification of asthma severity based on clinical features [20], as we previously reported [15]; two additional items asked about duration of asthma symptoms and physician-established diagnosis. 

### 2.3. Medical Records Review

Reviews were conducted by study physicians to extract information on established diagnoses of lung disease (such as allergic bronchopulmonary aspergillosis, chronic obstructive pulmonary disease, and interstitial lung diseases), on OSA diagnosis (which had been established by clinical polysomnography (PSG)), and whether or not CPAP was being used as documented in the clinic visit notes at the time of the survey. PSG indices or objective CPAP use were not collected in this study. Also, information was extracted on other comorbidities (e.g., GERD, rhinitis, chronic sinusitis, nasal polyps, and psychiatric diseases such as depression, anxiety, panic, or bipolar disorders) and current asthma medications, and from spirometry to help assess asthma step. 

The asthma step was assigned, according to NAEPP guidelines [20], as we previously reported [15], in order of increasing severity: (i) step 1 (mild-intermittent asthma), patients with daytime symptoms ≤2 days/week, nighttime symptoms ≤2 nights/month, Forced Expiratory Volume in first second (FEV_1_) ≥ 80% predicted and peak expiratory flow rates (PEFR) variability <20%; (ii) step 2 (mild-persistent asthma), patients with daytime symptoms 3–6 days/week, nighttime symptoms more than 2 nights/month (3-4 nights/month), FEV_1_ ≥ 80% and PEFR variability 20–30%; (iii) step 3 (moderate-persistent asthma), patients with daily asthma symptoms, nocturnal symptoms ≥5/month (or >once/week), FEV_1_ between >60%–<80% or PEFR variability >30%; (iv) step 4 (severe-persistent asthma), patients with continuous daytime symptoms, frequent nighttime symptoms, FEV_1_ ≤ 60% or PEFR variability >30%. The PEFR variability was calculated by dividing the difference between the highest and the lowest daily PEF value by the daily mean PEF and multiplying the result by 100 [21]. Attempts were made to obtain current peak flow diaries from patients or their medical records; due to their limited availability, for uniformity, the peak flow variability could not be used in asthma step assessment. The asthma step was determined by the most severe remaining qualifying features.

### 2.4. Data Analysis

Subjects were categorized into an older (60–75 years) or younger group (18–59 years). Baseline characteristics were summarized as mean ± standard deviation (SD) for continuous variables and percentages for categorical variables. The BMI was stratified per CDC criteria (<25, 25–29.9 and ≥30 kg/m^2^) and used as an ordinal variable. Occurrence of OSA symptoms “usually” or “always” (responses of 4 or 5 on the SA-SDQ) defined “habitual” symptoms. The validated cutoffs of SA-SDQ ≥36 for men and ≥32 for women defined high OSA risk [19]. Age at asthma onset was computed based on current age and duration of asthma symptoms, and age at physician-established diagnosis was recorded. Asthma step 3 or 4 defined severe asthma. To assess for any differential expression of relationships between daytime or nighttime asthma symptoms with OSA, daytime asthma symptoms occurring >2 days/week defined persistent daytime symptoms, and nighttime symptoms occurring >2 nights/month defined persistent nighttime symptoms.

The initial analysis was conducted on data from subjects without OSA and those with diagnosed OSA but not on CPAP treatment at the time of survey. Two-sample *t*-test, *chi*-squared, or Fisher exact test were used, as appropriate, to test for group differences in baseline variables. Ordinal logistic or logistic regression were used to test for univariate relationships of asthma step or severe asthma as the dependent variables with OSA diagnosis, BMI and traditional contributors to asthma control (demographics [age, gender and African-American race], rhinitis, chronic sinusitis, nasal polyps, GERD, and psychiatric disease) [14, 22]. The same techniques were employed to fit multivariate models with asthma step, severe asthma or persistent asthma symptoms as the outcomes, and OSA diagnosis as the predictor, while controlling for the above covariates, regardless of their univariate associations with these asthma outcomes. 

Secondly, data from subjects with diagnosed OSA were separately tested among each group, for associations of CPAP use with asthma step or severe asthma as the dependent variables. These multivariate logistic regression models were adjusted only for obesity, due to the small sample size of CPAP users, particularly in the older. 

 Statistical computations were conducted using SAS software Version 9.2 (SAS Institute, Cary, NC). A two-sided *P*-value <0.05 was required for statistical significance. Trends were noted when *P*-values ranged 0.05–0.10. 

## 3. Results 

Among 1,026 subjects invited to participate, 952 (93%) agreed and completed the survey. Of these, 64 subjects had comorbid lung disease and were excluded from the analyses. 

For the initial analysis, among 140 (36 older and 104 younger) subjects with previously diagnosed OSA, those 75 (16 older and 59 younger subjects) on treatment at the time of survey were excluded from analyses, as beneficial effects of CPAP treatment for OSA on asthma have been reported [12, 13]. Among the remaining 813 subjects, 154 (19%) were elderly and 659 (81%) young, among whom the main results are reported. The subsequent analysis encompassed solely the 140 subjects with diagnosed OSA, regardless of their treatment status at the time of survey. 

### 3.1. Phenotypic Characteristics of Older and Younger Asthma Subjects

Table 1 presents the demographic, physiologic, and clinical characteristics of the two groups. As compared to the young, older individuals had later onset of asthma symptoms and longer duration of physician-established diagnosis. They exhibited several indices of worse disease control, including physiologic measures and more frequent use of inhaled corticosteroid (ICS) at the time of survey. Older asthmatics had a higher prevalence of nasal polyps and GERD and a lower prevalence of psychiatric diseases. No statistically significant differences emerged in the other clinical variables presented (all *P* > 0.10).  Table 2 depicts the distribution of asthma severity step and severe asthma (step 3 or 4) among the older and young groups. As before, when accounting for symptoms, a tendency towards higher step category was seen in the older group, and a significantly larger proportion of older subjects (49% versus 39%, *χ* ^2^ = 5.53, *P* = 0.02) were in the severe asthma category. When evaluating the pattern of asthma symptoms, in comparison to the young, older subjects had similar frequencies of persistent daytime symptoms (31% versus 35%, *χ* ^2^ = 0.77, *P* = 0.38), but they less often had nighttime symptoms (29% versus 39%, *χ* ^2^ = 5.50, *P* = 0.02).

### 3.2. OSA Diagnosis and Symptoms in the Older and Younger Asthma Subjects

Table 3 presents the OSA diagnosis (clinically-diagnosed and untreated), SA-SDQ scores, and responses on its symptom-items. A larger proportion of older as opposed to younger subjects had OSA (13% versus 7%, *P* = 0.01); likewise, the mean SA-SDQ scores were higher in the older than in the young, and a higher proportion of older subjects (34% versus 25%, *P* = 0.03) achieved scores placing them at high risk for OSA, as defined by previously validated cutoffs [19]. Among individual SA-SDQ symptom items, witnessed apneas tended to be more prevalent in older patients who also had a significantly increased prevalence of current/history of hypertension; there was a lower prevalence of gasping arousals in the older relative to the younger patients.

### 3.3. Associations of Clinically-Diagnosed and Untreated OSA with Asthma Severity in the Older and Younger Asthma Subjects

Among older patients, in univariate ordinal logistic regression analyses (Table 4), statistically significant associations of asthma step with OSA and a history of GERD were noted, and trends were observed with BMI and rhinitis. In contrast, age, gender, African-American race, chronic sinusitis, nasal polyps, and a psychiatric history were not significantly associated with asthma step. Associations with OSA were of similar magnitude in the older and younger subjects. When examining severe asthma (Table 5), significant associations were also noted with OSA and GERD, and the relationship with OSA was of greater magnitude in the older than in the young.

In a multivariate model (Table 6) that included all the above variables as covariates, only OSA was significantly associated with asthma severity step: having an OSA diagnosis increased the odds of a worse asthma severity step on average by 191% (OR = 2.91, 95% confidence interval [1.15–7.36]), independent of the other characteristics. Associations of OSA with asthma step in older subjects seemed of greater magnitude than in the younger subjects. In a reiteration of this model using severe asthma as the dependent variable (Table 7), much more robust associations with OSA were observed in the older than in the younger subjects: independent of covariates, an OSA diagnosis raised the likelihood of severe asthma on average by 567% (OR = 6.67, 95% confidence interval [1.74–25.56]) in the older subjects versus 161% (2.61 [1.28–5.33]) in the younger subjects. 

### 3.4. Pattern of Asthma Symptoms and Their Associations with Clinically-Diagnosed and Untreated OSA in Older and Younger Asthma Subjects

A differential expression of the relationships between daytime or nighttime asthma symptoms with OSA was noted (Table 8): in the older subjects, nighttime asthma symptoms were primarily related to OSA (4.56 [1.55–13.43]), whereas in the younger subjects daytime asthma symptoms were associated with OSA (2.66 [1.32–5.32]), both after adjustment for the same variables shown in Tables 6 and 7.

### 3.5. Relationships of Asthma Severity with CPAP Use among Older and Younger Subjects with Clinically-Diagnosed OSA

Among clinically-diagnosed OSA subjects (*n* = 140), the frequency of CPAP use was not significantly different between the older and younger groups (21% versus 31%, *χ* ^2^ = 1.62, *P* = 0.20). In older subjects, with adjustment for BMI, CPAP use was associated with a reduced likelihood of worse asthma step by 86% (0.14 [0.04–0.56], *P* = 0.005) and of severe asthma by 91% (0.09 [0.02–0.49], *P* = 0.005); these relationships were of greater magnitude in older subjects than in younger subjects, where CPAP use attenuated the likelihood of worse asthma step by 58% (0.42 [0.20–0.88], *P* = 0.02) and that of severe asthma by 57% (0.43 [0.18–1.03], *P* = 0.06).

## 4. Discussion 

This analysis of a large clinic-based sample of patients with asthma shows for the first time that older as compared to younger asthmatics have worse asthma control and that poor control in older subjects may depend on comorbid OSA more than it does in younger subjects. Moreover, CPAP use attenuated the likelihood of worse asthma control, statistically, more robustly in older than in younger subjects. In marked contrast to OSA symptoms, traditionally recognized risk factors for asthma such as BMI, female gender, African-American race, rhinitis, and GERD showed little or no independent predictive value for asthma control among older persons. Collectively, these data suggest that underlying OSA may contribute to worse asthma control, particularly in the older patients, independent of other known asthma aggravators.

This clinic-based asthma sample is likely representative of older asthma patients, including those with unrecognized OSA, who are managed in specialty clinic settings. There was a high participation rate in our survey study (95%). Obesity rates were consistent with current estimates of obesity in this age group [23]. Mean duration of asthma was comparable with that reported in other studies [24]. Subjects demonstrated a higher FEV_1_/FVC ratio and much higher use of inhaled corticosteroids than what has been reported previously [2, 4], indicating appropriate use of treatment guidelines for asthma control in our sample. The high prevalence of patients with rhinitis (88%) reflects that this sample was recruited in good part from Allergy clinics. Rhinitis was not associated with asthma step in our population, likely due to its rigorous management by expert providers, which counteracted its detrimental influence on asthma control. Smoking was remarkably infrequent in our sample as compared to others [25], which may reflect a historical trend toward lower smoking rates or more effective education and interventions in two university clinic settings. Nonetheless, this finding supports the absence of confounding by comorbid chronic obstructive lung disease. 

Despite vigorous management, nearly half (49%) of our older subjects had worse asthma control (severe asthma: steps 3 and 4) as compared with 39% in the younger group, consistent with other reports [26]. This finding suggests that other factors for poor control of asthma, besides those usually recognized, may be at play. One explanation may be that older patients have worse physiological changes compared with younger asthmatics; however, no age effect was found in intraluminal obstruction and subepithelial collagen in a study of individuals who died from asthma [27]. Our data suggest an alternate explanation for this relationship.

We found a significant association of diagnosed OSA with worse asthma control (asthma step) among older patients. Associations of potential clinical significance have been identified between asthma and OSA, such that treatment for comorbid OSA improved asthma symptoms [12, 13, 28], use of rescue bronchodilator, peak expiratory flow rates [12], and disease-specific quality of life [29]. However, these data came from younger subjects, and to our knowledge, our study is the first to extend identification of OSA as a potential adverse contributor to asthma control to the aged. While asthma is an important cause of morbidity and mortality in older individuals, in regard to sleep and respiratory diseases in older persons, little data are available, pertaining mainly to disrupted sleep. Older patients with chronic airway obstruction, relative to controls, have a higher prevalence and greater severity of sleep difficulties and morning tiredness [10]. Furthermore, among older individuals with obstructive lung disease, sleep quality is worse in asthmatics than in individuals with COPD and chronic bronchitis [10].

For the first time, we report that the association of OSA with worse asthma control was of greater magnitude in older than in younger individuals (Tables 6 and 7) and, likewise, that CPAP use more robustly in the older than in the young attenuated the likelihood of worse asthma control. It is known that older adults are at increased risk for OSA and for more severe disease, which may be an explanation for our observation. Prevalence estimates for OSA among persons over 65 years of age, compared to those for the middle aged, are approximately 2-3 times higher [30]. In a large cohort of healthy older persons, the prevalence of OSA as defined by apnea hypopnea index (AHI) > 15 respiratory events/hr was 53%, and 37% had severe OSA (AHI > 30) [31]. Our sample demonstrates similar rates of snoring with a sample of 134 older subjects of similar age (mean 64.1 ± 9.1 years) and BMI (29.3 ± 5.3 kg/m^2^) [32]. Furthermore, in the most recent Wisconsin Sleep Cohort data, regardless of gender and BMI strata, the oldest group (50–70 years old) consistently demonstrated higher OSA (AHI ≥ 5 events/h) prevalence including that of clinically-significant (AHI ≥ 5 and daytime sleepiness) and of more severe (AHI ≥ 15 and daytime sleepiness) disease [33]. Age-related changes in the upper airway anatomy have been shown, including an increase in the soft palate length and parapharyngeal fat pads independent of BMI, as well as a decrease in the upper airway size [34, 35]. Aging also affects upper airway function by decreasing the genioglossus's response to negative pressure, a greater reduction in genioglossus and tensor palatini activity at sleep onset, and an increase in pharyngeal collapsibility and resistance independent of BMI and gender [34, 36, 37]. These effects may be compounded in older asthmatics by the effects of sleep on their lung properties—important determinants of upper airway patency during sleep [38]. There is an augmented decline in functional residual capacity (FRC) during sleep in patients with asthma: as compared to normal controls, among asthmatics, the FRC is significantly reduced while sleeping supine than during supine wakefulness. Loss of lung elastic recoil may reduce the effectiveness of lung volume stabilizing the upper airway during sleep [38]. Furthermore, in elderly asthmatics, an association between FRC (estimating degree of hyperinflation) and duration of asthma, as well as an inverse relationship of FEV_1_% predicted with duration of asthma has been shown; however, the former was independent of the degree of airflow limitation, suggesting that the duration of asthma is associated with both the degree of airflow limitation and hyperinflation [39]. This study suggested that with longer asthma duration, in time these abnormalities may become irreversible, as a reflection of distal airway and/or parenchymal changes as well as proximal airway remodeling [39]. Conversely, in regards to CPAP use, one predictor of beneficial effects of CPAP is the severity of disease. In one large study, the oxygen desaturation index (ODI) showed the strongest association with long-term adherence to CPAP; age was not a detrimental factor [40]. PSG in young asthma individuals of different severities and controls did find lower mean nocturnal SaO_2_ and higher AHIs with more severe asthma, compared with controls [41]. Therefore, a higher severity of disease, particularly its degree of hypoxemia in elderly asthma patients, could be a factor in their more reported benefit with CPAP. 

Another interesting and intriguing observation from this study is the differential pattern of associations of OSA observed with asthma symptoms: more with nighttime in older and more with daytime in younger individuals (Table 8). This has to be better understood in future studies, but one possibility remains that sleep in older patients with chronic airway obstruction being marked by more disruptions and awakenings [10] may render them more aware of their nighttime asthma symptoms than the young. 

How OSA can worsen asthma in general remains unknown, but there are several plausible pathways. The concept of a relationship between upper and lower airways called the *integrated airway* [42] is used to describe an inflammatory process within a continuous airway as opposed to distinct pathologies [43] and may also underlie the interaction of OSA with asthma. Repetitive upper airway obstruction results in intrathoracic pressure swings, frequent arousals, and intermittent hypoxemia that contribute to an inflammatory milieu, as demonstrated through associations with cardiovascular and cerebrovascular diseases [44, 45]. Other putative mechanisms include SDB-induced resistive loading [46] of an already challenged lower airways system during sleep [47], enhanced vagal tone during obstructive events [28], increased nonspecific bronchial reactivity [48, 49], altered chemical arousal thresholds [50], and upper airway occlusion [51], all of which may promote bronchoconstriction. Although experimental human studies of intermittent hypoxia are lacking, exposure to sustained hypoxia led to cough suppression [52] and impaired symptom perception in individuals with asthma [53]. OSA could also promote GERD [54], which is a well-recognized trigger of asthma [55].

Our data suggest a potential role of OSA in asthma control independent of other factors known to worsen this disease, such as obesity, particularly in the elderly [56, 57]. Studies of the relationship between obesity and asthma in older persons are scarce and do not account for OSA as a potential mediator, though obesity is an important risk factor for OSA [30]. In a population-based longitudinal study of older Norwegians, a U-shaped association between asthma and BMI was found [58]. However, our study adds new evidence to suggest that much of the association between obesity and asthma may in fact be explained by OSA. In our older subjects, accounting for OSA completely attenuated (Tables 6 and 7) the trends of BMI with asthma initially noted in univariate analyses (Tables 4 and 5). One other study that systematically assessed OSA syndrome in difficult-to-control asthmatics did report an increased risk for OSA (3.1 (1.1–9.0)), less severe airway obstruction, and eosinophilic inflammation and concluded that other factors, rather than airway inflammation alone, explain the relationship between obesity and asthma [59]. 

Among limitations to our study, we relied on the use of a questionnaire-based approach (SA-SDQ) to evaluate OSA symptoms prospectively and of previously clinically-documented diagnosis of OSA. Additionally, owing to lack of availability and heterogeneity of testing we did not have meaningful PSG data available. Costs of prospective PSG would have been prohibitive for an early-stage investigation of this size, and selection bias might well have been more prominent, as far fewer than 93% of approached patients would have agreed to participate. The SA-SDQ has been validated in a large sample of sleep patients with high internal validity, good sensitivity, and specificity [19]. Although lower threshold scores than those used herein have been validated in patients with epilepsy [60], application of those lower thresholds to patients with pulmonary fibrosis reduced the performance of the survey instrument, despite retention of a good correlation between SA-SDQ and OSA severity [61]. These data suggest that the original cutoffs [19], which we used in our study, may be more suitable for patients with chronic lung diseases. 

The cross-sectional design of this study limits conclusions about causality. Although asthma may be a risk factor for the development of OSA [62], our data fit with those from interventional studies [12, 13, 28, 29] and plausible mechanisms, as reviewed above, to suggest a detrimental effect of OSA on asthma control. Lastly, our sample included subjects aged up to 75 years, a restriction imposed by the design of the parent study. Our anecdotal observation conducting this survey is that there were quite small numbers of patients over 75 years of age attending our tertiary Allergy and Pulmonary clinics; therefore, we do not feel that this age restriction is likely to have had substantial impact on our findings. These patients may have unique comorbidity and cognitive profiles making them suitable for inclusion in future studies, specifically targeted to the older asthma patient population. 

In conclusion, this first study of asthma and its relationship with OSA among well-characterized patients finds in older individuals worse indices of disease control than in the young. It also shows associations of OSA diagnosis with worse asthma control indices that were independent of obesity and other recognized asthma aggravators and in great part attenuated by CPAP use. These associations were of greater magnitude in the older than in the younger subjects, suggesting that the former may draw greater benefit from treating OSA. These data are correlative and cannot prove causation, but they raise the possibility that in many cases, OSA may be an unrecognized contributor to asthma control in older persons. If so, identification and treatment of OSA may improve asthma control particularly in these patients.

## Figures and Tables

**Table 1:** Demographic and physiologic characteristics, medical history, and medication use for older and younger asthma subjects.

Characteristic	Mean ± SD, or Number (%) of Subjects		*P* value
	Older (*n* = 154)	Younger (*n* = 659)	
Age (y)	66 ± 4	42 ± 11	<0.0001
Gender (female)	95 (62%)	448 (68%)	0.14
BMI (kg × m^−2^)	29 ± 6	29 ± 7	0.36
<25	38 (25%)	214 (32%)	0.17
25–29.9	52 (34%)	204 (31%)	
≥30	64 (42%)	241 (37%)	
Race			0.65
African-American	7 (5%)	35 (5%)	
White	145 (94%)	598 (91%)	
Others*	2 (1%)	26 (4%)	
Current smokers	4 (3%)	33 (5%)	0.28
Age of asthma onset (y)	39 ± 21	22 ± 15	<0.0001
Age at physician-established asthma diagnosis (y)	41 ± 20	23 ± 16	<0.0001
FEV_1_% predicted	87 ± 19	94 ± 19	0.0002
FVC% predicted	84 ± 16	93 ± 16	<0.0001
FEV_1_/FVC	73 ± 9	77 ± 9	<0.0001
FEF_25–75_% predicted	56 ± 27	71 ± 31	<0.0001
History of rhinitis	137 (89%)	598 (91%)	0.50
History of chronic sinusitis	54 (35%)	192 (29%)	0.15
History of nasal polyps	32 (21%)	88 (13%)	0.02
History of GERD	84 (55%)	285 (43%)	0.01
History of psychiatric disease	29 (19%)	190 (29%)	0.01
Using inhaled corticosteroid (ICS)	132 (86%)	500 (76%)	0.008
Using oral corticosteroid	16 (10%)	54 (8%)	0.38
Using inhaled long-acting bronchodilator	97 (63%)	379 (58%)	0.22
Using antileukotriene agents	32 (21%)	182 (28%)	0.08
Using inhaled anticholinergic	10 (6%)	49 (7%)	0.69
Using theophylline	4 (3%)	13 (2%)	0.54

Definition of abbreviations: SD: standard deviation; BMI: body mass index; FEV_1_%: forced expiratory volume in first second; FVC%: forced vital capacity; FEF_25–75_%: forced expiratory flow between 25% and 75% of vital capacity (all of these physiologic variables are expressed as percentages of predicted values); GERD: gastroesophageal reflux disease.

*Included Asians, Hawaiian/Pacific Islander, and American Indians/Alaskans.

**Table 2:** Distribution of asthma severity step and severe asthma (step 3 or 4) among older and younger asthma subjects.

	Older (*n* = 154)	Younger (*n* = 659)	*P* value
	number (%)	number (%)	
Asthma step			
1	64 (42%)	312 (47%)	0.08
2	14 (9%)	90 (14%)	
3	44 (29%)	138 (21%)	
4	32 (21%)	119 (18%)	
Severe asthma	76 (49%)	257 (39%)	0.02

**Table 3:** OSA (clinically-diagnosed and untreated), SA-SDQ scores, and frequencies of individual symptoms (when present with any frequency), in older and younger asthma subjects.

	Mean ± SD or number (%) of subjects		*P* value
	Older (*n* = 154)	Younger (*n* = 659)	
OSA	20 (13%)	45 (7%)	0.01
SA-SDQ score	30.2 ± 6.4	27.7 ± 7.6	<0.0001
Snoring	126 (82%)	511 (78%)	0.25
Habitual snoring*	37 (24%)	172 (26%)	0.60
Witnessed apneas	50 (32%)	167 (25%)	0.07
Sudden gasping arousals	48 (31%)	276 (42%)	0.01
Excessive sweating at night	109 (71%)	481 (73%)	0.58
Current or history of hypertension	87 (56%)	182 (28%)	<0.0001
Nasal congestion during sleep	118 (77%)	485 (74%)	0.44
Snoring worse when supine	103 (67%)	433 (66%)	0.78
Snoring worse after alcohol	51 (33%)	236 (36%)	0.53

Definition of abbreviations: OSA: obstructive sleep apnea; SA-SDQ: Sleep Apnea scale of the Sleep Disorders Questionnaire (range 12–60); *Habitual snoring: snoring occurring “usually” or “always” (responses of 4 or 5 on the SA-SDQ).

**Table 4:** Univariate associations of asthma severity step with OSA (clinically-diagnosed and untreated) and other traditional factors of asthma control, in the older and younger asthma subjects.

	Older (*n* = 154)		Younger (*n* = 659)	
	Odds ratio [95% confidence interval]	*P* value	Odds ratio [95% confidence interval]	*P* value
OSA	3.44 [1.43–8.27]	<0.0001	3.61 [2.07–6.31]	<0.0001
Age	1.04 [0.97–1.11]	0.30	1.01 [1.00–1.02]	0.12
Gender (female versus male)	0.97 [0.54–1.77]	0.93	1.35 [1.00–1.83]	0.05
BMI (categorized)	1.37 [0.95–1.98]	0.09	1.84 [1.54–2.20]	<0.0001
African-American (versus all others)	1.90 [0.48–7.55]	0.36	4.04 [2.14–7.60]	<0.0001
Rhinitis	0.43 [0.17–1.09]	0.08	0.27 [0.17–0.44]	<0.0001
Chronic sinusitis	0.67 [0.36–1.23]	0.20	0.93 [0.68–1.28]	0.67
Nasal polyps	0.92 [0.45–1.88]	0.82	1.35 [0.90–2.04]	0.15
GERD	1.94 [1.08–3.51]	0.03	1.64 [1.23–2.18]	0.0007
Psychiatric disease	1.26 [0.60–2.62]	0.54	1.50 [1.10–2.04]	0.01

Definition of abbreviations: OSA: obstructive sleep apnea; BMI: body mass index (kg/m^2^); GERD: gastroesophageal reflux disease.

**Table 5:** Univariate associations of severe asthma (step 3 or 4) with OSA (clinically-diagnosed and untreated) and other traditional factors of asthma control, in the older and younger asthma subjects.

	Older (*n* = 154)		Younger (*n* = 659)	
	Odds ratio [95% confidence interval]	*P* value	Odds ratio [95% confidence interval]	*P* value
OSA	7.20 [2.02–25.74]	0.002	4.26 [2.19–8.28]	<0.0001
Age	1.04 [0.97–1.11]	0.30	1.01 [1.00–1.02]	0.12
Gender (female versus male)	0.97 [0.54–1.77]	0.93	1.35 [1.00–1.83]	0.05
BMI (categorized)	1.37 [0.95–1.98]	0.09	1.84 [1.54–2.20]	<0.0001
African-American (versus all others)	1.90 [0.48–7.55]	0.36	4.04 [2.14–7.60]	<0.0001
Rhinitis	0.43 [0.17–1.09]	0.08	0.27 [0.17–0.44]	<0.0001
Chronic sinusitis	0.67 [0.36–1.23]	0.20	0.93 [0.68–1.28]	0.67
Nasal polyps	0.92 [0.45–1.88]	0.82	1.35 [0.90–2.04]	0.15
GERD	1.94 [1.08–3.51]	0.03	1.64 [1.23–2.18]	0.0007
Psychiatric disease	1.26 [0.60–2.62]	0.54	1.50 [1.10–2.04]	0.01

Definition of abbreviations: OSA: obstructive sleep apnea; BMI: body mass index (kg/m^2^); GERD: gastroesophageal reflux disease.

**Table 6:** Multivariate associations of asthma severity step with OSA (clinically-diagnosed and untreated) and other traditional factors of asthma control, in the older and younger asthma subjects.

	Older (*n* = 154)		Younger (*n* = 659)	
	Odds ratio [95% confidence interval]	*P* value	Odds ratio [95% confidence interval]	*P* value
OSA	2.91 [1.15–7.36]	0.02	2.07 [1.14–3.75]	0.02
Age	1.03 [0.96–1.10]	0.48	1.00 [0.99–1.02]	0.89
Gender (female versus male)	0.89 [0.46–1.74]	0.74	1.45 [1.06–1.99]	0.02
BMI (categorized)	1.22 [0.82–1.81]	0.32	1.60 [1.32–1.93]	<0.0001
African-American (versus all others)	0.76 [0.17–3.43]	0.72	3.08 [1.60–5.93]	0.0008
Rhinitis	0.48 [0.19–1.25]	0.14	0.36 [0.22–0.59]	<0.0001
Chronic Sinusitis	0.60 [0.29–1.24]	0.17	0.88 [0.62–1.26]	0.49
Nasal Polyps	1.34 [0.55–3.26]	0.52	1.26 [0.79–1.95]	0.33
GERD	1.58 [0.86–2.92]	0.14	1.22 [0.89–1.67]	0.22
Psychiatric disease	1.33 [0.60–2.97]	0.48	1.31 [0.94–1.82]	0.11

Definition of abbreviations: OSA: obstructive sleep apnea; BMI: body mass index (kg/m^2^); GERD: gastroesophageal reflux disease.

**Table 7:** Multivariate associations of severe asthma (step 3 or 4) with OSA (clinically-diagnosed and untreated) and other traditional factors of asthma control, in the older and younger asthma subjects.

	Older (*n* = 154)		Younger (*n* = 659)	
	Odds ratio [95% confidence interval]	*P* value	Odds ratio [95% confidence interval]	*P* value
OSA	6.67 [1.74–25.56]	0.006	2.61 [1.28–5.33]	0.008
Age	1.06 [0.98–1.14]	0.17	1.01 [0.99–1.02]	0.45
Gender (female versus male)	1.32 [0.61–2.86]	0.49	1.48 [1.03–2.11]	0.04
BMI (categorized)	1.06 [0.68–1.65]	0.81	1.49 [1.20–1.84]	0.0003
African-American (versus all others)	1.89 [0.27–13.24]	0.52	4.26 [1.89–9.59]	0.0005
Rhinitis	0.40 [0.12–1.28]	0.12	0.45 [0.25–0.81]	0.007
Chronic sinusitis	0.38 [0.16–0.91]	0.03	0.81 [0.54–1.22]	0.31
Nasal polyps	1.73 [0.60–4.96]	0.31	1.32 [0.78–2.23]	0.30
GERD	1.35 [0.67–2.71]	0.41	1.30 [0.91–1.85]	0.16
Psychiatric disease	1.68 [0.65–4.33]	0.28	1.05 [0.72–1.54]	0.81

Definition of abbreviations: OSA: obstructive sleep apnea; BMI: body mass index (kg/m^2^); GERD: gastroesophageal reflux disease.

**Table 8:** Multivariate associations of persistent daytime and nighttime asthma symptoms with OSA (clinically-diagnosed and untreated) with adjustment for age, gender, race (African-American versus. all others), rhinitis, chronic sinusitis, nasal polyps, gastroesophageal reflux disease, and psychiatric disease, in the older and younger asthma subjects.

	Older (*n* = 154)		Younger (*n* = 659)	
	Odds ratio [95% confidence interval]	*P* value	Odds ratio [95% confidence interval]	*P* value
Persistent daytime symptoms	2.00 [0.71–5.65]	0.19	2.66 [1.32–5.35]	0.006
Persistent nighttime symptoms	4.56 [1.55–13.43]	0.006	1.35 [0.70–2.63]	0.37
